# The spatial phenotype of genotypically distinct meningiomas demonstrate potential implications of the embryology of the meninges

**DOI:** 10.1038/s41388-020-01568-6

**Published:** 2020-12-01

**Authors:** Daniel M. Fountain, Miriam J. Smith, Claire O’Leary, Omar N. Pathmanaban, Federico Roncaroli, Nicoletta Bobola, Andrew T. King, Dafydd Gareth Evans

**Affiliations:** 1 Geoffrey Jefferson Brain Research Centre, Salford Royal NHS Foundation Trust and the University of Manchester, Manchester, UK; 2 Manchester Centre for Genomic Medicine, Manchester Academic Health Sciences Centre (MAHSC), St Mary’s Hospital, School of Biological Sciences, Division of Evolution and Genomic Sciences, University of Manchester, Manchester, UK; 3 School of Medical Sciences, Faculty of Biology, Medicine and Health, University of Manchester, Manchester, UK

**Keywords:** Cancer genetics, Differentiation

## Abstract

Meningiomas are the most common primary brain tumor and their incidence and prevalence is increasing. This review summarizes current evidence regarding the embryogenesis of the human meninges in the context of meningioma pathogenesis and anatomical distribution. Though not mutually exclusive, chromosomal instability and pathogenic variants affecting the long arm of chromosome 22 (22q) result in meningiomas in neural-crest cell-derived meninges, while variants affecting Hedgehog signaling, PI3K signaling, *TRAF7*, *KLF4*, and *POLR2A* result in meningiomas in the mesodermal-derived meninges of the midline and paramedian anterior, central, and ventral posterior skull base. Current evidence regarding the common pathways for genetic pathogenesis and the anatomical distribution of meningiomas is presented alongside existing understanding of the embryological origins for the meninges prior to proposing next steps for this work.

## Introduction

**Fig. 1:**
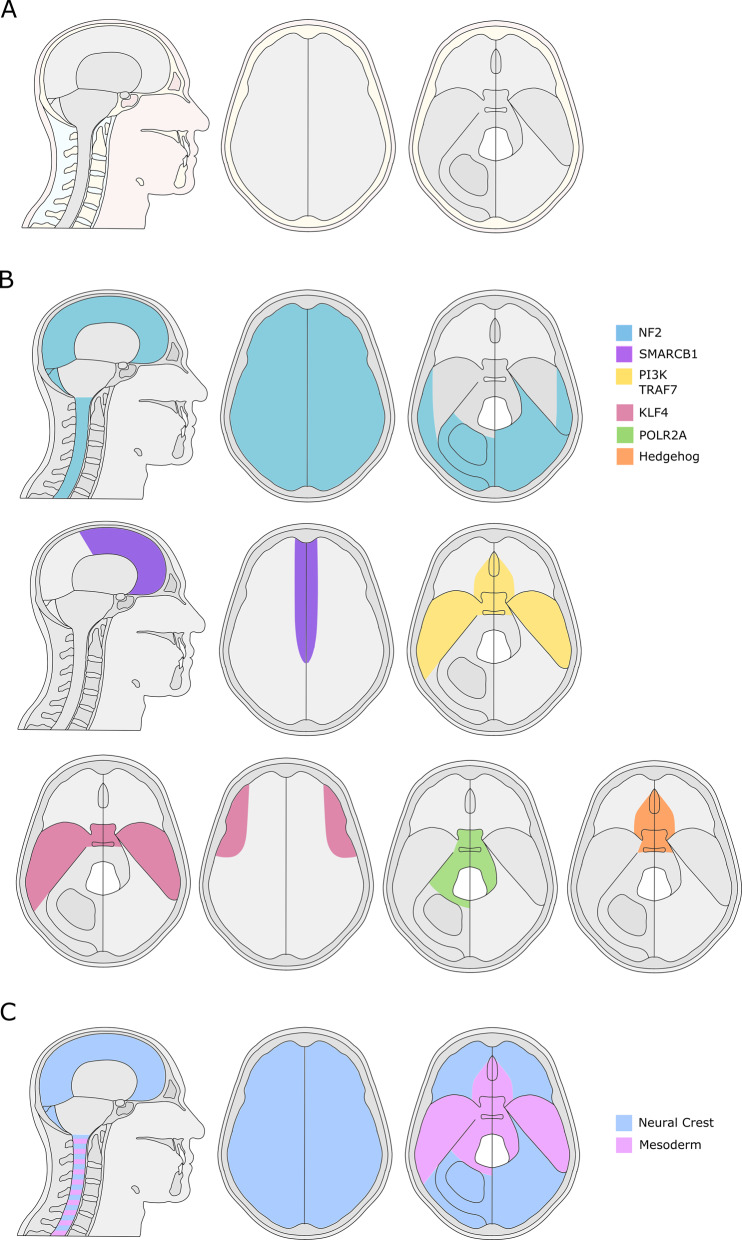
The spatial phenotype of genotypically distinct meningioma and embryology of the meninges. **A** Anatomical depiction of meninges with brain and spinal cord removed displaying skull base, sagittal, and convexity regions including tentorium cerebelli on the right side. **B** distribution of meningioma by pathogenic variant gene pathway. **C** meningeal embryonic development by the tissue of origin.

Meningiomas are the most common primary brain tumor, representing 37% of all intra-cranial tumors with an annual incidence of 4.5 per 100,000 people with a lifetime risk of around 1 in 280, and their incidence and prevalence is increasing [1–3]. Incidence over a 14-year period (1999–2013) of diagnoses and surgical resection of meningiomas have increased by 52% and 58% respectively [4]. Skull base meningiomas represent up to half of all meningiomas requiring surgery [5]. Due to their proximity to cranial nerves, brainstem, upper cervical spinal cord, and critical cerebral vasculature, they are challenging to resect completely (Fig. 1); consequently recurrence rates can be as high as 29% [6, 7]. Around a third of recurrences are of a higher tumor grade (World Health Organization (WHO) grade II and III [8]. Patients with atypical (WHO grade II) and malignant (WHO grade III) meningioma suffer from a high morbidity and mortality, with reported 10-year survival of 63% and 15% respectively, in spite of a relatively young mean age at diagnosis [1, 4]. Aside from radiotherapy which has a limited evidence base, there are scarce alternative therapies with proven efficacy [9].

The cell of origin of a meningioma is frequently reported to be the arachnoid cap cell, primarily due to cytological similarity [2]. It is however more probable meningiomas develop both from dural border cells and arachnoid barrier cells based on the shared expression of prostaglandin D2 synthase (PGDS) in these cellular layers and meningiomas [10, 11]. This may also explain the broad spectrum of histologically distinct variants in the classification of meningiomas, which remain classified solely according to histological appearances (Table 1) [2]. Robust epidemiological data of the incidence of meningioma by histological subtype has not been reported. Based on genomic data from a recently published cohort, meningothelial (41%) and transitional (17%) subtypes represent the most common variants [12]. A study of registry data reported 80.6% of meningioma were WHO grade I, 17.6% WHO grade II, and 1.7% WHO grade III [1].

**Table 1:** Proportion of meningioma by histological subtype (*n* = 1970).

WHO grade	Histological subtype	Proportion/% [12]
I	Meningothelial	41.2
I	Fibrous (fibroblastic)	12.6
I	Transitional (mixed)	16.5
I	Psammomatous	2.9
I	Angiomatous	2.2
I	Microcystic	3.8
I	Secretory	3.8
I	Lymphoplasmacyte-rich	0.2
I	Metaplastic	0.3
II	Chordoid	0.7
II	Clear cell	0.4
II	Atypical^a^	13.8
III	Papillary	0.1
III	Rhabdoid	0.2
III	Anaplastic^b^	1.4

Reproduced from Youngblood et al. [12].

^a^Criteria: brain invasion, mitotic count 4–19/10 HPF (high-power field), 3 of the following: spontaneous necrosis, loss of whorling or fascicular architecture, prominent nucleoli, high cellularity, and high nuclear to cytoplasmic ratio.

^b^Criteria: overtly malignant cytology, 20 or more mitoses/10 HPF.

Cranial meningiomas most commonly develop in the convexity and parasagittal regions and in the skull base in relation to the sphenoid (Table 2) [13]. Registry data from the USA reported that 79.8% of meningiomas were located in the cranial meninges compared to 4.2% located in spinal meninges (the remainder were unknown) [1]. In an alternative study of 25,694 surgically treated meningiomas in England, 92.3% were located in the cranial meninges compared to 7.7% located in the spinal meninges [4].

**Table 2:** Proportion of meningioma by location (*n* = 1113).

Location	Proportion/% [5]
Skull base	51.5
Anterior cranial fossa	13.7
Middle cranial fossa	17.2
Posterior cranial fossa	20.7
Convexity/falx/parasagittal	48.5
Falx/parasagittal	19
Convexity	19.7
Other	9.8

Reproduced from Magill et al. [5].

Neurofibromatosis type 2 (*NF2*) is the most common and first identified driver gene associated with meningioma. *NF2* encodes the protein Merlin, which was initially found to interact with CD44 during contact inhibition of cell proliferation, but additionally inhibits PI3K/mTORC1/Akt signaling pathways and activates the mammalian Hippo pathway [14]. This has traditionally been associated with convexity meningiomas in patients with *NF2*, a tumor suppressor syndrome where patients have a 50–75% lifetime risk of developing a meningioma [15–17]. However, pathogenic variants in *NF2* have been identified in 40–60% of sporadic meningiomas [2]. Genomic analysis of *NF2* and non-*NF2* sporadic meningiomas have identified further pathogenic variants in *AKT1* [18], *AKT3* [19], BRCA1-associated protein 1 (*BAP1)* [20, 21], Kruppel-like factor 4 (*KLF4*) [18], *PIK3CA* [22], *PIK3R1* [19], *POLR2A* [19], *PRKA-R1A* [19], *SMARCB1* [23–25], subfamily B, member 1 (*SMARCE1*) [26], smoothened (*SMO*) [27], *SUFU* [28], and TNF receptor associated factor 7 (*TRAF7*) [18]. Epidemiological molecular data are lacking; based on the largest genomic study to date, *NF2* remains the most commonly affected gene in meningiomas (Table 3) [12].

**Table 3:** Proportion of meningioma by causative gene (*n* = 1970).

Gene	Proportion/% [12]
*NF2*	38.3
*MU*	26.1
*PI3K*	12.8
*TRAF7* alone	5.5
*HH*	4.8
*SMARCB1*	2.5
*KLF4*	7.6
*POLR2A*	2.4

Reproduced from Youngblood et al. [12].

*MU* mutation unknown, *HH* Hedgehog pathway genes.

This review presents existing evidence of the relationship between the histological subtype, WHO grade, and genomic alterations of meningioma and the location of their development. The embryology of the meninges is presented, summarizing the hypothesis that the cephalic mesoderm contributes to the meninges of the midline and paramedian ventral posterior and central skull base. A synthesis is summarized including evidence of meningioma pathogenesis through interruption of genes in key developmental pathways. Implications for the utility of therapies used in other tumor types, development of in vitro and in vivo modeling of meningioma genetic pathogenesis, and patient selection for trials, are discussed.

## Meningioma and its location—genomics and histology

With the advent of next-generation sequencing, significant advances have been made in the last decade in identifying pathogenic variants in meningioma tumorigenesis. These have been categorized into major gene pathways which demonstrate striking mutual exclusivity across multiple studies [3, 19]. These are summarized in Table 4.

**Table 4:** Summary of genomic, histological, and locational characteristics of meningioma by identified gene pathway [12, 18, 19, 21, 22, 24, 27, 29–34].

Category	Gene	WHO	Histology	Pathogenic variant	Location
22q deletion	*NF2*	I-III	Fibrous	Multiple	Falx cerebri
			Psammomatous		Cerebral and cerebellar convexity
			Transitional		Tentorium cerebelli
			Atypical		Spinal
			Anaplastic		
	*SMARCB1*	I-III	Fibrous	Multiple	Anterior falx cerebri
			Transitional		
			Atypical		
Hedgehog signaling	*SMO*	I	Meningothelial	p.Leu412Phe	Anterior medial skull base
				p.Trp535Leu	
	*SUFU*	I	Meningothelial	p.Arg123Cys	
	*PRKA-R1A*	I	Meningothelial	p.Ala17Asp	
PI3K pathway	*PIK3CA*	I	Meningothelial	p.Glu545	Anterior and middle medial skull base
			Transitional	p.His1047	
	*PIK3R1*	I	Meningothelial	Multiple	
	*AKT1*	I	Meningothelial	p.Glu17Lys	
			Transitional		
	*AKT3*	I	Meningothelial	p.Glu17Lys	
Other	*TRAF7*	I	Meningothelial	Multiple	Anterior and middle medial skull base
			Secretory		
	*KLF4*	I	Secretory	p.Lys409Gln	Middle and lateral skull base
	*POLR2A*	I	Meningothelial	p.Gln403Lys	Middle and posterior medial skull base
				p.Leu438_His439del	
	*BAP1*	II	Rhabdoid	Multiple	Insufficient evidence
	*SMARCE1*	II	Clear cell	Multiple	Insufficient evidence

### 22q deletion (*NF2*, *SMARCB1*)

Pathogenic variants in *NF2* are associated with somatic loss of the second chromosome 22 allele [27, 29, 30], and are strongly though not exclusively associated with fibrous, psammomatous, transitional, atypical, and anaplastic meningioma. [27, 31–33] *NF2*-mutated meningioma constitute the majority of meningiomas located in the falx cerebri, tentorium cerebelli, and cerebral and cerebellar convexities [12, 18, 32]. In large-scale genomic studies of meningioma, higher grade (WHO grade II and III) meningioma were in some studies exclusively related to pathogenic variants in *NF2*, associated with mutations in the *TERT* promoter, and deletion of 1p and *CDKN2A* [30, 34]. SWI/SNF related, matrix associated, actin dependent regulator of chromatin, *SMARCB1*, adjacent to *NF2* on chromosome 22q, has been identified to contribute to meningioma tumorigenesis with somatic missense mutations identified in exon 9 [24], and a germline variant in exon 2 [35]. In patients with NF2, those with truncating *NF2* mutations towards the 5′end of the gene were associated with a higher prevalence and lifetime risk of meningioma [15]. A four-hit mechanism has been proposed resulting in tumor suppressor gene inactivation and the development of familial multiple meningiomas [25]. It is of interest that few variants associated with *SMARCB1* related schwannomatosis have been associated with meningioma risk and overall the chances of developing meningioma in *SMARCB1* related schwannomatosis without these specific missense variants is low [23, 36].

### PI3K-AKT-mTOR pathway (*AKT1*, *AKT3*, *PIK3CA*, *PIK3R1*)

Pathogenic variants in AKT serine/threonine kinase 1 (*AKT1*), an oncogenic component of the (PI3K)-AKT-mTOR pathway, have been identified in non-NF2 meningiomas of the medial skull base [18, 27, 37]. Subsequent studies have additionally identified an association of *AKT1* pathogenic variants with benign WHO grade I meningothelial subtype lacking genomic instability [18, 30, 34]. Mutually exclusive somatic pathogenic variants have additionally been identified in *AKT3* [19], phosphatidylinositol-4,5-bisphosphate 3-kinase (PI3K), the catalytic subunit alpha (*PI3KCA*) and regulatory subunit alpha (*PI3KR1*) have also been identified in meningiomas with WHO grade I meningothelial or transitional histology arising from the medial skull base [19, 22].

### Hedgehog signaling pathway (*SMO*, *SUFU*, *PRKA-R1A*)

*SMO*, a G-protein coupled receptor and key transmembrane protein member of the Hedgehog signaling pathway, was identified in several studies of non-NF2 meningiomas [18, 27]. SUFU (suppressor of fused homolog) protein acts downstream of SMO and loss of *SUFU* function has been implicated in familial multiple meningioma [28]. Pathogenic variants in *PRKA-R1A* have additionally been identified in a small proportion of meningiomas [19]. *PRKA-R1A* is a critical component of type I protein kinase A (PKA) and pathogenic variants result in increased PKA activity and subsequently increased SMO cell surface accumulation thus contributes to Hedgehog signaling [19, 38]. Meningiomas harboring pathogenic variants in the Hedgehog signaling pathway are more likely to develop as a WHO grade I meningothelial subtype in the midline anterior fossa floor of the skull base [12, 18, 27]. Across multiple studies it has been additionally demonstrated that meningiomas with pathogenic variants in the Hedgehog signaling pathway are not associated with genomic instability [18, 30, 34].

### Other pathogenic variants (*KLF4*, *TRAF7*, *SMARCE1*, *BAP1*)

*KLF4* is a transcription factor known to induce pluripotency in adult fibroblast cultures [39]. The role of *KLF4* is context-specific, with evidence of its function both as an oncogene and tumor suppressor in cancer [40]. A highly recurrent p.Lys409Gln mutation was identified in the first of three zinc fingers pivotal for DNA binding [41]. Meningiomas with pathogenic variants involving *KLF4* were more commonly identified in the skull base away from the midline [12]. Secretory meningiomas have been defined based on combined pathogenic variants of *KLF4* and *TRAF7* mutually exclusive of the PI3K pathway or *NF2* [41]. Hallmarks of secretory meningioma, hyaline periodic acid-Schiff-positive globules, and peritumoral edema, are suspected mechanistically to be associated to *KLF4* signaling as a result of regulatory of cytokeratins 4 and 19 and activation of the bradykinin B2 receptor [41].

*TRAF7* has been reported as the most common pathogenic variant identified in non-NF2 meningioma [18, 19, 22]. Pathogenic variants in have been reported to occur in combination with *AKT1*, *PIK3CA*, *PIK3R1*, and *KLF4* [12, 18, 19, 41]. Multiple somatic mutations were identified in an intronic hot spot of *TRAF7* related to the first WD40 domain which plays an important regulatory role in the NF-κB pathway [18, 42]. Meningiomas with TRAF7 pathogenic variants alone or in combination commonly develop in the skull base, with isolated TRAF7-mutated meningioma associated with a microcystic histological subtype [12].

Recurrent pathogenic variants in polymerase (RNA) II (DNA directed) polypeptide A (*POLR2A*) are characterized by mutations localized to the dock domain involved in formation of the pre-initiation complex [19]. Meningiomas with identified pathogenic variants in *POLR2A* are most commonly mutually exclusive, genomically stable, and associated with benign meningiomas in the midline skull base, in particular the region of the tuberculum sellae [19].

*BAP1* is involved in the response to DNA damage as a tumor suppressor gene functioning as a ubiquitin carboxy-terminal hydrolase [21]. Germline mutations in *BAP1* result in a cancer syndrome involving the development of BAP1-mutated melanocytic skin tumors and a high incidence of mesothelioma [43]. All tumors share a common histological rhabdoid morphology. Of the six tumors with *BAP1* mutations and BAP1 loss on immunohistochemistry, four were located in the convexity regions and two in the skull base [21]. There is currently insufficient evidence to demonstrate a spatial phenotype of these tumors.

SWI/SNF related, matrix associated, actin dependent regulator of chromatin, *SMARCE1* pathogenic variants have been specifically associated with heritable clear cell meningiomas [26, 44, 45]. Initially suspected to present exclusively as multiple spinal meningiomas [26], cases of cranial meningiomas with pathogenic variants in *SMARCE1* have subsequently been identified [44]. Clear cells are characterized by vacuolated cytoplasm and bland nuclei in a whorled, syncytial architecture, a likely consequence of SMARCE1 protein loss [46]. While the histology is diagnostic of WHO grade II clear cell subtype, of the few cases reported they have included meningiomas of the spine, convexity, and skull base regions without a propensity to a specific location [45]. A simplified summary of the above pathogenic variant categories and their relationship with histological subtype and the location of meningioma tumorigenesis is shown in Fig. 1B.

## Meningioma and its location—embryological origin of the meninges

The most comprehensive early study of the development of the meninges was undertaken by O’Rahilly and Muller in 1986 [47]. This study of cranial meninges involved the serial sectioning of 61 human embryos. At Carnegie stage (hereafter stage) 11 (24 postovulatory days), the pia mater is first identified at the caudal medulla while elsewhere a thick mesenchyme surrounds the developing brain. This thick mesenchyme is derived from a combination of neural crest cell mesoectoderm and neurilemmal cells, the prechordal plate, the unsegmented paraxial mesoderm, and the segmented paraxial (somitic) mesoderm. By stage 15 (33 postovulatory days) this mesenchyme surrounds most of the brain and is called the primary meninx. Subsequently, the primary meninx differentiates into the pachymeninges (later dura mater) and leptomeninges (later arachnoid and pia mater) [47, 48]. Similar work was undertaken by Sensenig in characterizing the embryological origin of the spinal meninges, where paraxial somitic mesodermal and neural crest cells were concluded to contribute to the dura and arachnoid mater (mesodermal) and pia mater (neural crest), respectively [49].

The development of quail-chick chimeras resulted in the ability to track the migration of neural crest cells, demonstrating the contribution of the neural crest to the meninges of the forebrain while the meninges of the brainstem derive from cephalic mesoderm [50, 51]. HNK1 expression was used to further demonstrate a contribution of the neural crest to the spinal meninges, in contrast with earlier studies demonstrating an exclusively mesodermal contribution [52, 53].

The use of permanent molecular markers for neural crest cells and developmental stage-specific conditional knockout mice has resulted in significant progress with characterization of the embryonic origin of the cranial bones and meninges [10, 54–57]. The use of X-gal staining and Dil labeling has been used in transgenic mice with in vivo permanent labeling of neural crest and mesoderm (Wnt1-Cre/R26R and Mesp1-Cre/R26R strains, respectively) [54, 56]. A further PGDS transgenic Cre strain was developed based on PGDS representing a specific marker of arachnoidal cells. The PDGS positive meningeal cell was identified as a common precursor to both the dural border cells and arachnoid border cells [11, 58]. Collectively, these models have demonstrated that the meninges at the skull base derive from mesoderm, while the meninges covering the cerebral and cerebellar hemispheres derive from the neural crest [10, 54, 55, 59].

Most recently, single-cell transcriptomic analyses of meningeal fibroblasts in the forebrain have identified fibroblast populations that are transcriptionally distinct between brain regions, particularly in pia mater [60]. The authors state that anterior meninges arise from the neural crest, whereas posterior meninges originate from the mesoderm, and conclude that due to this mixed contribution there is regionalization of gene expression. Of particular relevance is the M3 subcluster, an arachnoid cell cluster, where in the embryonic day 14.5 (E14.5) mouse embryo in in situ validation there was patchy expression of *Ptgds*, the gene encoding PGDS, in the dorsal telencephalon contrasting with high expression throughout the skull base surrounding the midbrain and hindbrain regions [60].

Notable similarities have been identified between the development of the meninges and the skull bones. Animals with mutations in *Foxc1*, an identified gene crucial in meningeal development, develop significant meningeal and calvarial defects [61, 62]. Furthermore, intramembranous ossification of mesodermal bone requires interaction with neural crest-derived meninges [56, 63]. In the transgenic mouse models, the frontal, ethmoid, presphenoid, squamous temporal, and interparietal bones were identified as neural crest derived. Conversely, parietal, non-squamous temporal, and basioccipital bones are derive from mesoderm [55, 56, 59]. The middle of the basisphenoid, corresponding to the sella turcica in the adult skull base, marks the demarcation between bone derived from neural crest and mesoderm with the notable exception of the post-optic root of the presphenoid bone which is derived from mesoderm [55, 64].

The identified junction at which cranial bones are derived from mesoderm and neural crest are species-specific, and it is probable that this is also observed in the meninges [65]. Overall, the likely development of the adult human meninges is a complex interplay between neural crest and mesodermally derived cells resulting in differentiation into cytologically similar meninges in the adult (Fig. 1).

## Meningioma genetic pathogenesis and embryological development—a synthesis

The above evidence demonstrates a clear, reproducible correlation between the location of a meningioma and the types of underlying pathogenic variants identified as driving tumorigenesis. There is evidence that the spatial contribution of the mesoderm and neural crest to the meninges correlates with the locations of commonly identified pathogenic variants in meningioma. In molecular profiling of 86 sequenced, spatially distinct meningiomas, expression of neural crest genes have been implicated in meningeal tumorigenesis [66], suggesting that meningioma tumorigenesis capitalizes on gene regulatory networks with subsequent misactivation of a developmental cell population [67, 68]. However, there still remains a great deal that is unknown; in the largest study of 1970 meningiomas with targeted and/or whole exome sequencing, 667 (26.1%) did not have an identified mutation [12]. While mechanistic explanations have now been provided for the development of multiple histological subtypes, the underlying genomic characteristics of microcystic grade I and chordoid grade II meningioma also remain unknown [12, 69]. This section reviews the underlying mechanisms of tumorigenesis in the meninges and its relationship to developmental pathways.

### The neural crest, *NF2*-Hippo, and the SWI/SNF complex

The development of neural-crest-derived tissues is dependent on a process balancing proliferation, migration, and pluripotency that share many characteristics with tumorigenesis [70]. The development of *NF2* knockout mice resulted in greater understanding of the role of the gene during development. *NF2* null mice die during embryonic development due to a failure to initiate gastrulation [71], while heterozygous models resulted in widespread tumor development [72], and conditional *NF2* gene inactivation in leptomeningeal cells resulted in the development of meningiomas [73]. The use of a β‐gal reporter under the control of an *NF2* promoter in transgenic mice identified intense β‐gal staining in forebrain and telencephalon extending caudally in the later covered by pia mater, consistent with meningeal layers derived from the neural crest and the most common locations for the development of *NF2*-mutated meningioma [74].

Merlin is known to have multiple functions, but with respect to meningioma pathogenesis it notable for its role as a tumor suppressor regulating proliferation and apoptosis through Hippo signaling [75], and in cellular motility, spreading and attachment through mediation of the actin cytoskeleton [76]. Yap, a component of the Hippo pathway and inhibited upstream by Merlin, has been implicated in neural crest cell fate and migration [77, 78]. *SMARCB1* loss in the early neural crest results in the development of human rhabdoid tumors, while induced loss at a later stage results in Schwannomatosis [79]. Overall, the SWI/SNF complex is strongly linked to mammalian differentiation and is a critical regulator of pluripotency in embryonic stem cells, with *SMARCB1* essential for neural induction but non-essential for mesodermal differentiation [67, 80].

The relationship between Hedgehog signaling and meningioma pathogenesis is perhaps the most convincing. In a zebrafish model, Hedgehog signaling is required for cranial morphogenesis and chondrogenesis in the midline of the zebrafish skull [81]. Dysregulation results in craniofacial defects including holoprosencephaly and hypotelorism [82]. *SMO*-mutated meningiomas occur predominantly in the midline anterior skull base. Conditional activation of *SMO* in developing mouse embryos resulted in the development of meningothelial meningiomas in the ventral skull base, with similar location and histological appearances to *SMO*-mutated meningioma [83].

The role of the cranial neural crest and mesoderm in craniofacial development is not mutually exclusive, with interdependence identified in the patterning of facial tissues and chondrogenesis [56, 63, 84]. Manipulation of migratory and proliferative behaviors reveals crucial interactions between the two cell populations in normal embryogenesis. Although a simplification, on review of the embryological origin of the meninges, there is reason to hypothesize that the relative contribution of cell types to different layers and regions of the meninges may contribute to an explanation for these spatial phenotypes.

## Future directions

Existing in vitro models use highly malignant immortalized meningioma cells that do not represent the diverse genomic characteristics of meningiomas, particularly of the skull base [33]. Challenges exist due to the senescence of in vitro cell lines of benign meningiomas [2]. The different developmental progenitors of meninges of the skull base should be considered in any future meningioma models including pathogenic variants commonly found in this region. Pathogenic variants associated with specific histological subtypes will likely be incorporated into future classification guidance, and it is recommended that location is included as part of this. Where whole genome sequencing is not possible for every patient, targeted sequencing of pathogenic variants corresponding to the location of the resected meningioma will be crucial to facilitate participation in trials with targeted therapy and for prognostic information for patients. The development of a molecularly driven trial of patients given targeted therapy based on their *AKT1*, *SMO*, and *NF2* pathogenic variant status is an ideal example of the future for clinical trials in patients with meningioma (NCT02523014) [85].

There are still conflicting accounts regarding the cell of origin of the meningioma, with arachnoid cap cells, arachnoid barrier cells and dural border cells candidates. There has been limited consideration to tumor heterogeneity although limited unpublished evidence suggests this could be substantial [66]. The tumor microenvironment should be examined in the context of extended understanding of the heterogeneity of meningiomas, and the gene regulatory networks underlying meningeal development given its correlation with the genomic signatures of meningiomas. DNA methylation profiling has been used successfully to predict risk of recurrence and prognosis across multiple studies, and demonstrates the significance of epigenetic modifications in tumor pathogenesis, particularly given the relative lack of chromosomal instability in meningiomas of the skull base [30, 86]. Capitalizing on spatial and temporal transcriptomics will facilitate greater understanding in both animal models and tumor samples of remaining candidate pathogenic variants responsible for meningioma pathogenesis and identify developmental pathways that could be modulated resulting in more effective targeted therapies for these tumors.

With the advent of immunotherapy, understanding of the tumor microenvironment has become pivotal in identifying potential immune-mediated mechanisms for treatments. Understanding of the tumor microenvironment in meningioma is comparatively understudied, with no existing single-cell transcriptomic immune cell profiling currently. T cell repertoire characterization of 28 meningiomas of all grades identified populations of CD4+ and CD8+ T cells, regulatory T cells, and T cells expressing PD-1 (Programmed cell death protein 1) indicative of exhaustion [87]. A study of bulk transcriptomic data from 107 meningiomas identified immune processes to be the sole biological mechanism correlated with anatomical location after correcting for the WHO grade in the tumor [88]. Whereas oncolytic gamma-delta T cells dominate skull base meningiomas, mast cells and neutrophils were more prominent in convexity meningiomas [88]. Conversely, a study of tumor-associated macrophage infiltration in meningioma found no significant differences in the macrophage number or ratio of M1 to M2 phenotype between the skull base and convexity meningioma samples [89].

Given the increasing importance of location in the understanding of tumor biology and immune microenvironment, biologically and clinically meaningful and accurate classification is essential. Despite the numerous surgical classifications of meningioma subtypes [90–93], and classifications in studies including genomic characteristics [12], there is currently no international consensus regarding the reporting of a location of a meningioma, particularly classification of meningiomas of the skull base. Reaching a consensus will facilitate cross-study comparisons and drive standardization in the investigation and reporting of meningioma pathogenesis.

## Conclusions

In summary, there is emerging evidence of a correlation between location, phenotype and genotype in meningioma and such correlation has its basis on the embryology of meninges. A combination of temporal and spatial epigenetic and genetic analyses is required to better characterize the developing meninges, the arachnoid from which meningiomas are thought to derive, and meningiomas themselves to advance our understanding of these tumors for further biomarker and therapy discovery and implementation.
