# Correction: The spatial phenotype of genotypically distinct meningiomas demonstrate potential implications of the embryology of the meninges

**DOI:** 10.1038/s41388-021-01700-0

**Published:** 2021-09-14

**Authors:** Daniel M. Fountain, Miriam J. Smith, Claire O’Leary, Omar N. Pathmanaban, Federico Roncaroli, Nicoletta Bobola, Andrew T. King, Dafydd Gareth Evans

**Affiliations:** 1 Geoffrey Jefferson Brain Research Centre, Salford Royal NHS Foundation Trust and the University of Manchester, Manchester, UK; 2 Manchester Centre for Genomic Medicine, Manchester Academic Health Sciences Centre (MAHSC), St Mary’s Hospital, School of Biological Sciences, Division of Evolution and Genomic Sciences, University of Manchester, Manchester, UK; 3 School of Medical Sciences, Faculty of Biology, Medicine and Health, University of Manchester, Manchester, UK

**Keywords:** Cancer genetics, Differentiation

Correction to: *Oncogene*

10.1038/s41388-020-01568-6

In the original published version the “Funding statement” was incorrect. Please find the correct “Funding statement” below.

The original article has been corrected.

